# Microsatellite mutations in buccal cells are associated with aging and head and neck carcinoma

**DOI:** 10.1038/sj.bjc.6604198

**Published:** 2008-01-22

**Authors:** R J C Slebos, M Li, S Vadivelu, B B Burkey, J L Netterville, R Sinard, J Gilbert, B Murphy, C H Chung, Y Shyr, W G Yarbrough

**Affiliations:** 1 Department of Cancer Biology, Vanderbilt-Ingram Cancer Center, Vanderbilt University School of Medicine, Nashville, TN 37232, USA; 2 Department of Otolaryngology, Vanderbilt-Ingram Cancer Center, Vanderbilt University School of Medicine, Nashville, TN 37232, USA; 3 Department of Biostatistics, Vanderbilt-Ingram Cancer Center, Vanderbilt University School of Medicine, Nashville, TN 37232, USA; 4 Division of Hematology/Oncology Department of Medicine, Vanderbilt-Ingram Cancer Center, Vanderbilt University School of Medicine, Nashville, TN 37232, USA

**Keywords:** microsatellite, mutation, aging, carcinogen exposure, head and neck squamous cell carcinoma

## Abstract

Carcinogen exposure from tobacco smoking is the major cause of upper aerodigestive tract cancer, yet heavy smokers only have about a 10% life-time risk of developing one of these cancers. Current technologies allow only limited prediction of cancer risk and there are no approved screening methods applicable to the general population. We developed a method to assess somatic mutational load using small-pool PCR (SP-PCR) and analysed mutations in DNA isolated from cells obtained by mouth rinse. Mutation levels in the hypermutable tetranucleotide marker D7S1482 were analysed in specimens from 25 head and neck squamous carcinoma (HNSCC) cases and 31 controls and tested for associations with age, smoking history and cancer status. We found a significant association between mutation frequency and age (*P*=0.021, Generalized Linear Model (GLM), *N*=56), but no influence of smoking history. Cases had higher mutation frequencies than controls when corrected for the effects of age, a difference that was statistically significant in the subgroup of 10 HNSCC patients who were treated with surgery only (*P*=0.017, GLM, *N*=41). We also present evidence that cancer status is linked to levels of nonunique, and presumably clonally derived, mutations in D7S1482. Insertion mutations were observed in 833 (79%) of 1058 alleles, of which 457 (43%) could be explained by insertion of a single repeat unit; deletion mutations were found in 225 (21%) of tested alleles. In conclusion, we demonstrate that the sensitive detection of single molecule mutations in clinical specimens is feasible by SP-PCR. Our study confirms an earlier report that microsatellite mutations increase with age and is the first to provide evidence that these mutations may be associated with cancer status in individual subjects.

It is estimated that exposure to tobacco carcinogens causes up to 90% of respiratory tract cancers (ACS, 2003), yet the risk of developing a tobacco-related cancer in a lifetime smoker is less than 10%. Currently, the only useful measures to predict an individual's risk for developing lung or head and neck cancer are based on exposure, age of the individual and, if detectable, the presence of premalignant lesions. Given the significant role of carcinogen exposure in these tumours, it proves difficult to study host factors related to cancer risk (Proia *et al*, 2006). Such studies are based on measurement of host DNA repair capacity, such as the bleomycin chromosome breakage assay (Wu *et al*, 1998; Rajaee-Behbahani *et al*, 2001), the host-cell reactivation assay (Diem and Runger, 1997), or the comet assay (Kleinsasser *et al*, 2000). These assays suggest host impairments in DNA repair, but they have found limited application in the clinical setting because they require intact cells, are labour intensive and may have limitations with respect to reproducibility (Berwick and Vineis, 2000).

Mutations in microsatellites provide an alternative and more direct avenue to determine susceptibility to DNA damage. The benefit of such an approach lies in the possibility that differences in carcinogen metabolism may more accurately predict cancer risk. Microsatellites consist of repetitive DNA sequences (Weber, 1990) that can pose difficulties for cellular DNA replication and repair mechanisms (Gordenin and Resnick, 1998). In cells with intact MMR, microsatellite repeat sequences are sensitive to mutagen-induced mutations, first shown in an *Escherichia coli* model system (Jackson *et al*, 1998). Low levels of microsatellite mutations have been observed in tumours with no known mutations in mismatch repair genes, such as those of the lung, bladder, and head and neck (Mao *et al*, 1994; Shridhar *et al*, 1994; Woenckhaus *et al*, 2003). Mutations in lung cancer are more frequently found in tetranucleotide repeats than in dinucleotide repeats (Spafford *et al*, 2001; Xu *et al*, 2001), a phenomenon called Elevated Microsatellite Alterations at Selected Tetranucleotide repeats (EMAST). The underlying mechanism for EMAST is not known, but it is possible that mechanisms other than MMR gene deficiency contribute to the mutations in these tumours, especially in selected microsatellites with AAAG or ATAG repeat units (Ahrendt *et al*, 2000; Spafford *et al*, 2001; Xu *et al*, 2001). In one study, the occurrence of (AAAG)_n_ mutations was associated with mutations in the p53 tumour suppressor gene (Ahrendt *et al*, 2000).

In human lung or head and neck tumours, microsatellite mutations might reflect the high mutational load experienced by the epithelial lining of the respiratory tract exposed to tobacco smoke. We previously demonstrated that microsatellite repeat sequences are more sensitive to mutagens than nonrepeated sequences, leading to frameshifts in a reporter construct (Slebos *et al*, 2002), while a study of primary lung tumours in patients who had undergone previous radiation and/or chemotherapy treatment for Hodgkin lymphoma showed increased levels of microsatellite mutations (Behrens *et al*, 2000). Some tetranucleotide repeats also demonstrate high levels of instability in the germline (Ellegren, 2000). Results from these studies suggest that microsatellites are highly mutable, making them an attractive target for study, and that tetranucleotide repeats may be particularly sensitive to DNA damage. Technical advances have resulted in high-throughput methodology that allowed us to study microsatellite mutations at the single molecule level in clinical specimens (Coolbaugh-Murphy *et al*, 2004).

Mutation measurements are complicated by a process called ‘field cancerisation’, by which carcinogen exposure of a larger field of cells (e.g. oral or bronchial epithelium) leads to groups of related, but histologically normal, cells that spread to cover a large epithelial surface (Slaughter *et al*, 1953). We, and others, showed that such premalignant groups of cells can carry distinct genetic alterations that are identical to those found in overt cancers (Sozzi *et al*, 1995; Westra *et al*, 1996; Mao *et al*, 1997; Tabor *et al*, 2001). In our study, field cancerisation may lead to an apparent increase in the mutation measurement due to selective outgrowth of an otherwise normal cell clone carrying a mutant microsatellite sequence.

We here describe a study of mutations in the tetranucleotide marker D7S1482 in cells obtained by mouth rinse in a case–control study for head and neck cancer. We report a strong correlation between microsatellite mutations and age, but not with smoking history, and that cancer cases have higher age-controlled mutation levels than controls, suggesting that microsatellite mutations may be useful as markers for cancer risk.

## METHODS

### Clinical specimens and sample processing

All specimens were obtained from the Head and Neck Tissue Repository at Vanderbilt University with subjects consenting according to IRB-approved protocols. Cases were identified by searching all incident HNSCC patients seen at Vanderbilt between 2003 and 2006 who had completed curative treatment and who had a mouth rinse collected during a follow-up visit and no evidence of disease based on clinical exam at time or collection or during the follow-up period (median follow-up time 29 months, range 6–40 months). Control subjects were recruited during screening days for head and neck cancer organised through the Yul Brynner Foundation during 2004 and 2005 (www.headandneck.org). None of the control subjects had any symptoms of HNSCC on clinical exam. An effort was made to cover the full range for age and smoking histories, and to match cases and controls based on these parameters. In the final study set, the median age difference between cases and matched controls was 0 years (range: 13–14 years) with 19 of 25 pairs matched within 5 years of age. Matching by sex and by smoking history (ever *vs* never) was successful for 20 of 25 case–control pairs each.

Subjects donated a blood specimen, a mouth rinse and answered a simple questionnaire on demographic, clinical and exposure information. Mouth rinse was collected using an over-the-counter mouth wash and kept at 4°C until processing. The cells were collected by centrifugation at 1200 **g** and DNA was isolated using the Gentra Puregene buccal cell kit (Qiagen, Valencia, CA, USA). Germline allele sizes were tested in blood lymphocyte DNAs and found to be in accordance with the most frequently observed allele sizes in all buccal cell DNAs.

### Analysis of microsatellite mutations

Microsatellite mutations were detected similarly to the method described by Coolbaugh-Murphy *et al* (Coolbaugh-Murphy *et al*, 2004), with the main difference of using a single amplification reaction using time-release PCR (Slebos *et al*, 2004b). To determine the appropriate dilution levels we ran test amplifications with 10-fold dilution series on all DNAs. Traditional DNA quantitation was of limited value for this purpose presumably because of varying levels of nonhuman DNA in the mouth rinse samples. A wide variation in DNA concentration was observed between different preparations from cells obtained by mouth rinse, with optimal dilutions ranging from 8 to 50 000-fold of the initial DNA preparation. Experiments that had on average more than four molecules per well in a 96-well plate, as determined by counting blank wells and Poisson modeling (see statistical analysis below), were excluded to retain sensitivity to detect mutant sequences.

To assess variability of ‘small-pool’ PCR (SP-PCR) measurements we analysed duplicate D7S1482 measurements on identical DNA preparations from 23 of the subjects described in this study (Supplementary Table 1). We formally tested for differences between first and second replicate analysis using the Poisson-based GLM and this test demonstrated no significant difference in number and type of mutations between the measurements.

### Microsatellite marker analyses

We report mutation analysis results obtained with the tetranucleotide microsatellite marker D7S1482, which is a hypermutable marker in both germline and somatic cells (Temam *et al*, 2003; Slebos *et al*, 2004a). DNAs obtained from mouth rinses was diluted to near-single molecule levels and amplified using time-release PCR (Kebelmann-Betzing *et al*, 1998; Slebos *et al*, 2004b). The resulting SP-PCR reflects the amplification of small numbers of molecules that can be separated by automated capillary gel electrophoresis as reported by others (Coolbaugh-Murphy *et al*, 2005). Details of the method have been described previously (Slebos *et al*, 2004b). The optimal dilution for each DNA was determined by testing serial dilutions of a small number of tubes and estimating DNA concentration as described under statistical analysis below. In addition to D7S1482, we also used markers MycL1 and DXS981 to more accurately determine DNA quantity for single-molecule analysis.

**Figure 1:**
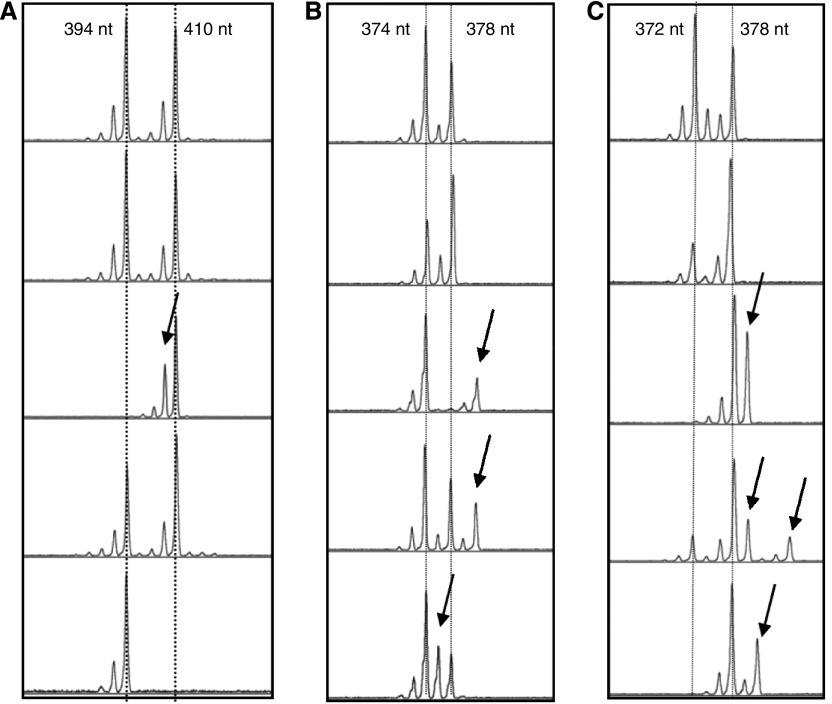
Example of microsatellite analysis. DNA from cells obtained by mouth rinse from three subjects were analysed by SP-PCR using the marker D7S1482 (**A**–**C**). Each subpanel represents the results from one PCR tube with 5 subpanels shown for each subject. The normal (nonmutated) fragment length is indicated by the dotted line, mutant DNA fragments are indicated by arrows.

Mutations were scored according to the following criteria. Insertion mutations were scored when peaks of at least 20% the height of the wild-type peak closest to the presumed mutant peak was observed in a position that indicated increased fragment length and that did not correspond to the peaks observed in undiluted DNAs obtained from blood lymphocytes of the same subject (for example Figure 1C, third panel). Similarly, deletion mutations were scored as peaks of at least 50% of the height of the nearest wild-type peak in the spectrum indicating decreased fragment length compared to the germline pattern (for example Figure 1A, middle panel). These criteria are conservative since germline allelic patterns judged according to these criteria never resulted in a mutant score. Mutations were assumed to originate from the germline fragment size closest to the one observed for the aberrant signal. Aberrant signals in positions more than three repeat units away and signals equidistant to either germline fragment sizes were scored as ‘other’ mutations (Figure 1B bottom panel and 1C 4th panel).

### Statistical analysis of microsatellite data

We studied the association between mutant frequency of D7S1482 and the *a-priori* defined parameters of age, smoking status and case–control status. With the assumptions that the event of observing a mutant allele is independent between subjects and the rate of mutation is constant over time, the number of mutant observed has a Poisson distribution given by 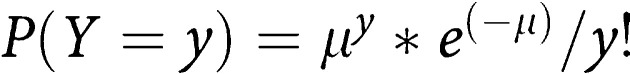 where *y* is the number of mutant alleles observed and *μ* is the expected count of mutant alleles and this expected value, *μ*, is the product of the mutant frequency and the total number of alleles. A Generalised Linear Model (GLM), Poisson Regression, can be adopted to model the effects of the above clinical parameters on microsatellite mutation frequency. However, it is not appropriate to compare the expected number of mutant alleles when the total number of alleles at risk may differ for each subject in different treatment groups. Therefore, the Poisson model will include an offset equal to the log of the total number of alleles tested and model the mutation frequency that can be compared between subjects in different groups.

Applying a GLM would be straightforward if we have the data with the number of mutant and the total number of alleles at risk for mutation. However, it was not possible to count the total number of alleles directly from each experiment and this value needs to be estimated. This was accomplished by estimating the average number of alleles per well in a 96-well plate, since the total number of alleles is the product of the average number of alleles per well and the number of wells (fixed). We derived the average number of alleles per well from the 96-well plate by recording the number of nonamplifying wells (blank wells) for either D7S1482 or a combination of D7S1482, DXS981 and MycL1. The number of blank wells from a total 96 wells follows a binomial distribution with a ‘success’ probability *p* that is the probability of zero alleles observed in the well. The count of alleles also has a Poisson distribution with 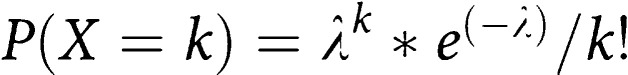, where *k* is the observed number of alleles per well, *λ* is the expected (average) number of alleles per well. Obviously, we have the ‘success’ probability *p* from the binomial distribution that equals the Poisson probability for k equals 0, that is, 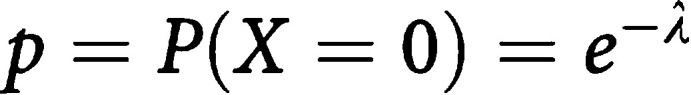. Since the marker measurements are independent, we can get the likelihood function that is the product of binomial mass function for the marker(s), and this function can be expressed in terms of *λ*. The maximum likelihood estimator MLE of *λ* can then be obtained by maximising this likelihood function. After estimating the average number of alleles per well, the total number of alleles can be calculated as the product of this maximum likelihood estimator of *λ* and the number of wells. In this calculation of *λ*, we take into account the differences between males and females in copy numbers of the X-chromosome linked marker DXS98 and markers MycL1/D7S1482/DXS981 exist in a 2 : 2 : 1 ratio in males and in a 1 : 1 : 1 ratio in females. With the estimated total number of alleles and the number of mutant observed, we applied the GLM to examine the effects of age, smoking and case status on mutation frequencies. We considered significance at the 0.05 level.

## RESULTS

### Study subjects and demographic information

A total of 56 subjects (25 cases and 31 controls) were studied for D7S1482 microsatellite mutations in cells from oral rinses (Table 1). The age distribution and smoking histories of cases and controls was very similar although there were proportionally more females in the control group than in the case group. Since samples from cases were obtained after cancer treatment, DNA damage due to therapy with radiation or chemotherapeutic drugs is a potential confounder favoring higher mutation measurements in cases compared to controls. On the other hand, cancer treatment may selectively eliminate abnormal cells that might be mutant for D7S1482, which would have the opposite effect on mutation frequency measurements. Confounding by treatment is taken into account in the statistical analyses by including these treatments in the model and by a separate analysis with the 10 patients who were not treated by chemotherapy and/or radiation therapy (eight oral cavity and two larynx carcinoma patients).

### Types of mutations as study end points

The primary end point was D7S1482 mutation frequency, defined as the summation of all mutations observed in D7S1482 divided by the total number of alleles tested. Such mutations can be explained by at least two mechanisms: (1) independent mutational events occurring in individual cells; and (2) linked mutational events arising from clonal outgrowth of a single cell containing the microsatellite mutation. We presume that at least a subset of mutations observed more than once in a single specimen are not independent in origin, which violates the assumption of independence in the statistical model. One solution to this problem is to ignore any mutations that occur two or more times and scoring only the number of mutant positions that are observed in a given sample. We will call this secondary end point ‘unique mutations’. This measure is less sensitive than overall mutation frequency since some mutations detected more than once may be the result of independent events rather than clonal outgrowth, but using this end point assures that the observed unique mutations are truly derived from independent events. On the other hand, mutant alleles arising from clonal outgrowth of normal mucosal cells are of interest in upper aerodigestive tract cancers, since these potentially clonal mutations may represent a measure of field cancerisation. Any mutant allele that is observed more than once may result from an identical independent mutational event or from clonal outgrowth of a ‘cancerised’ normal mucosal cell. Since we cannot distinguish between these two possibilities we will define these potentially clonal mutations as ‘nonunique mutations’. In a setting where a single allele-size is observed multiple times, nonunique mutation frequency is expected to capture at least a proportion of mutations from a clonal outgrowth of normal mucosal cells.

### Microsatellite mutations, age and smoking

**Figure 2:**
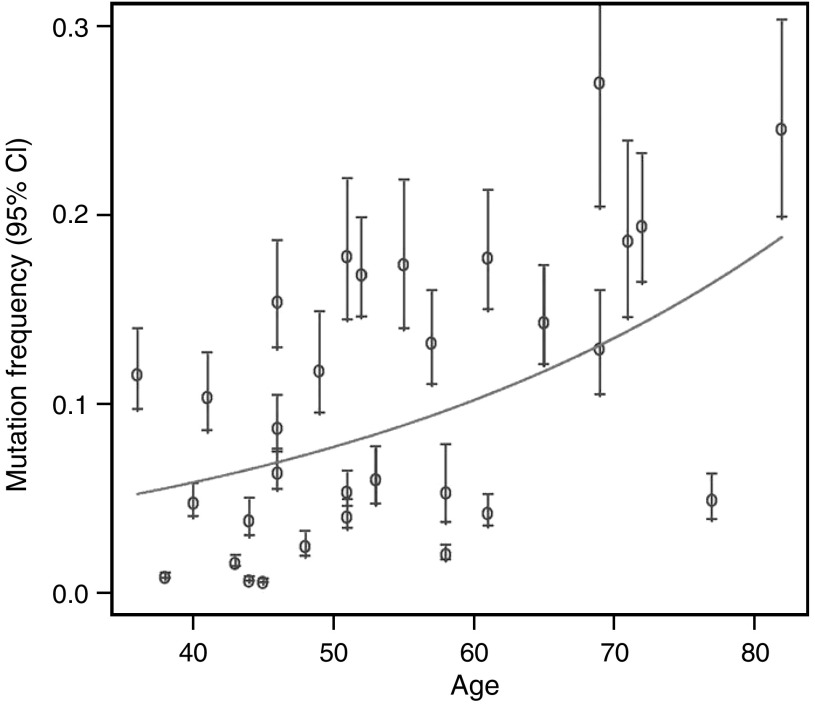
Observed and expected mutation frequency and age. Indicated are the observed D7S1482 mutation frequencies with 95% CI for all 31 control subjects. Mutation frequency increases with increasing age (*P*=0.004, GLM) as indicated by the predicted estimate from the univariate statistical model (solid line).

Based on a previous study on microsatellite mutations in blood lymphocyte DNA (Coolbaugh-Murphy *et al*, 2005), we performed a univariate analysis of the association of microsatellite mutations with age in the control group. As shown in Figure 2, univariate analysis showed a statistically significant increase of D7S1482 mutation frequency with increasing age (*P*=0.004, GLM). Although there was a clear trend towards increasing mutation frequency with increasing age, substantial individual differences were also observed. Since cells obtained by mouth rinse are potentially exposed to carcinogens from tobacco smoke, we next included smoking in the model, either as pack-year (PY, number of packs per day multiplied by number of years smoked) or categorised as never, former or current smoker. These comparisons did not demonstrate any effect of smoking although the effect of aging remained significant. Similarly, there were no differences in direct comparisons between the three classes of smokers (never *vs* ever, never *vs* current, never *vs* former or former *vs* current).

### Cancer status and microsatellite mutations

Differences in D7S1482 mutation levels between HNSCC cases and controls were tested by a generalised linear model (GLM), Poisson regression model, including all cases and controls with age, pack-year and case status as covariates. In this model, only the effect of age was significant (*P*=0.021, GLM), whereas cases had higher mutation levels but this did not reach statistical significance (*P*=0.104, GLM) (Table 2). For example, the rate ratio per 10-year increase in age is about 1.34 with 95% confidence interval 1.05–1.70, indicating that the increase in mutation rate per 10-year interval is about one third. When all subjects are considered, the rate ratio for HNSCC cases *vs* controls is 1.61 (95% CI 0.91–2.82). However, the analysis of all patients in the study is complicated by the fact that 15 of the 25 cases either received radiation and/or chemotherapy prior to collection of the mouth rinse specimens. We therefore limited our analysis to the 10 cases treated with surgery only and compared these to the full control group. In this analysis, the effect of age was borderline significant (*P*=0.067, GLM), while cases had significantly higher levels of D7S1482 mutations than controls (*P*=0.017, GLM). In this comparison, surgery-only HNSCC cases had on average twofold higher mutation frequencies than controls (95% CI 1.17–3.67) (Table 2).

### Unique and nonunique microsatellite mutations

When we tested unique mutations as end point, the effects of aging remained strong but again, no apparent effect of smoking could be discerned. In the control group, the GLM including age and smoking, age was significantly correlated with increasing unique mutations (*P*=0.036, GLM). As with overall mutations, neither PY nor smoking status had any significant effect in this model. Next, we tested the effect of cancer status in a model that included age, smoking and case–control status using unique mutations in D7S1482 as end point. In this comparison, age remained highly significant (*P*=0.002, GLM), but smoking (as PY or by category) or case status did not show any effect (Table 2). A similar result was obtained when the 10 surgery-only cases were used in this comparison.

As previously discussed, levels of nonunique microsatellite mutations in cells obtained by mouth rinse may indicate clonal outgrowth of normal mucosal cells and hence be a measure of field cancerisation within the oral cavity. Using nonunique mutations as end point, we tested for the effect of aging and smoking using the control group only (Table 2). Levels of nonunique mutations again increased with age (*P*=0.035, GLM) but not with pack-year (*P*=0.443, GLM). We then tested for increases associated with case status in a model including all cases and controls using D7S1482 as end point (Table 2). In this model, the increase in mutations with age was statistically significant (*P*=0.045, GLM) but smoking again did not show any significant difference. Case status was borderline significantly associated with higher nonunique mutation levels (*P*=0.091, GLM) but in the comparison with the 10 surgery-only patients an increase in nonunique mutations was statistically significant (*P*=0.012, GLM).

### Mutation patterns in D7S1482

**Figure 3:**
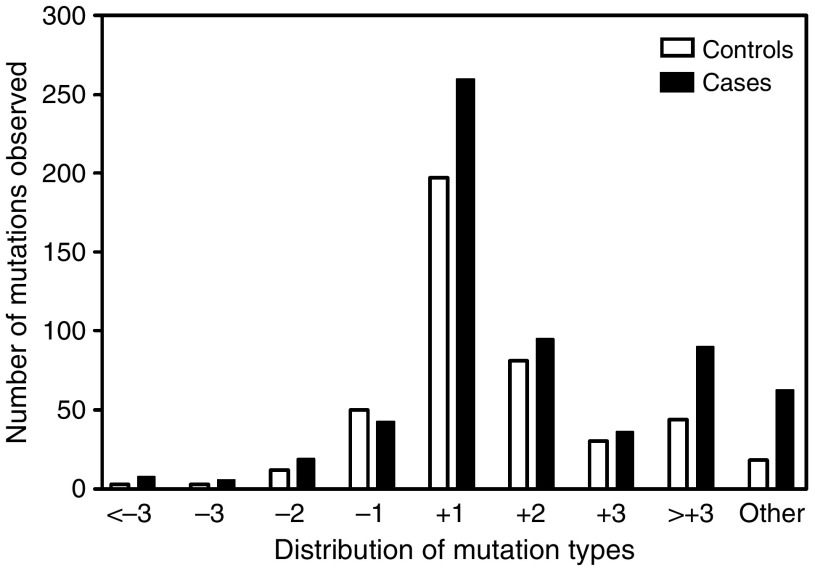
Distribution of insertion and deletion of repeat-unit mutations among all 1058 observed mutations. Approximately 40% of all mutations could be explained by a simple insertion of a single repeat-unit in one of the germline alleles. Insertion mutations were more frequently observed than deletion mutations (79 *vs* 21%). No obvious differences were seen between cases and controls.

Mutations in D7S1482 were scored according to the minimal frame-shift that would explain the observed mutant alleles. We observed a total of 1058 mutant alleles across all subjects studied. The majority of these mutant alleles were insertion mutations: 833 (79%) of 1058 alleles were larger than the size of the corresponding germline allele, of which 457 (43%) could be explained by the insertion of one repeat unit (four nucleotides) 176 (17%) by two repeat units, 66 (6%) by three repeat units and the remaining 134 (13%) by four or more repeat units or by undetermined repeat-unit shifts. Deletion mutations were much less often observed, 225 alleles were smaller than their corresponding germline alleles. The type of mutations in D7S1482 did not appear to be different between HNSCC cases and controls (Figure 3) although cases had a higher number of mutations classified as ‘other’ compared to controls (63 *vs* 18). However, 32 of these mutations were derived from one case with closely spaced germline alleles that did not allow us to distinguish between a −1 or +1 repeat unit mutation and hence were scored as ‘other’ mutations.

## DISCUSSION

Cancers of the upper aerodigestive tract are one of the most common cancers, but despite recent advances in genomics and proteomics, there are only a limited number of biomarkers for individual cancer risk. The accessibility of the oral mucosa allows noninvasive sampling of cells exposed to environmental carcinogens. The study of genetic changes in these exposed cells may elucidate mechanisms of carcinogenesis or identify candidate biomarkers to determine risk of upper-airway cancers. Our study is the first to investigate a possible use of a highly unstable tetranucleotide marker in relation to age, smoking and HNSCC using small-pool PCR on cells obtained by mouth rinse. We observed a highly significant correlation of mutation frequencies with the subject's age, a correlation that was stable in several of the markers and subject subgroups studied. These results are in agreement with the elegant study by Coolbaugh-Murphy *et al* (Coolbaugh-Murphy *et al*, 2005) where levels of microsatellite mutations measured in blood lymphocytes also correlated with age. Apart from a difference in target cells, these authors used a different panel of microsatellite markers that included 1 mono-, 4 di- and 1 trinucleotide markers, but not any tetranucleotide repeats. However, in our experience mononucleotide repeats, such as BAT26 and BAT25, are very difficult to score for mutations due to slippage artefacts during PCR, which renders these markers less sensitive for true mutations. The same problem, although less pronounced, also occurs when testing dinucleotide markers. An additional problem associated with some microsatellite markers is that of allelic dropout, where amplification reactions do not yield any DNA products. Allelic drop-out may be due to high levels of recombination resulting in deletion of the marker, variant sequences around the marker that disrupt PCR amplification or other causes. We previously found that the tetranucleotide markers CSF1R-CITT and D21S1245 had high levels of nonamplifying alleles and similarly Coolbaugh-Murphy *etal* (2005) reported this phenomenon for the dinucleotide marker D17S250. In the current study, we did not observe any allelic dropouts when determining germline allelic patterns. In terms of methodology, our strategy allows small-pool PCR using a single-tube PCR setup, facilitated by time-release PCR (Slebos *et al*, 2004b), that is amendable to robotic automation. This setup reduces sample handling and thus the risk of PCR contamination.

In previous studies, 25–50% of HNSCCs showed abnormalities in D7S1482 (Coulet *et al*, 2000; Temam *et al*, 2005). In one of these studies, the majority of the mutations observed were insertion mutations (Coulet *et al*, 2000). In addition, D7S1482 has highest level of mutations among all EMAST markers (five of 18) HNSCC and five of 47 NSCLC, again, mostly by insertion mutations (Xu *et al*, 2001). Since we have to assume the absence of selection pressure on this noncoding microsatellite, these data suggest that high levels of mutations in precancerous cells become fixed during clonal outgrowth of a mutant cell (field cancerisation) (Tabor *et al*, 2001).

Recently, several studies on the mechanisms that underlie differences in mutation rates between markers and the association with aging were reported. For example, the tetranucleotide marker MycL1 was found to be more stable than other microsatellites in MMR-negative cells, but more unstable in MMR-positive cells (Hatch and Farber, 2004). Kovtun *et al* (2007) demonstrated age-dependent expansion of a human Huntington's disease-related trinucleotide in transgenic mice). The levels of expansion were greatly increased in double-transgenic mice that lacked a single base-excision repair enzyme, 7,8-dihydro-8-oxoguanine-DNA glycosylase (OGG1), indicating the important role of oxidative damage in the expansion process. This study confirms and expands our knowledge that oxidative DNA damage can lead to microsatellite mutations (Jackson *et al*, 1998; Zienolddiny *et al*, 2000; Slebos *et al*, 2002). Other types of DNA damage, such as those caused by ionising radiation, chemotherapeutic drugs, or exposure to cadmium (Slebos *et al*, 2002; Jin *et al*, 2003; Kendall *et al*, 2004) may also lead to mutant microsatellite sequences. With the large number of studies suggesting that DNA damage leads to increased levels of microsatellite mutations it is unclear why we failed to observe a clear relationship between microsatellite mutations and smoking history in our study. This was not due to lack of range of smoking exposures in our control group (see Table 1). Some possible explanations might be that D7S1482 does not capture the effects of smoking exposure or that pack-year is linked to age to the extent that, with a limited study size, we lacked the power to detect an effect of smoking. Larger study sets with additional markers are needed to address this issue.

Of interest, the distinction of unique *vs* nonunique mutations, although not perfect, separated associations with aging and cancer status. Unique mutations were most strongly associated with age, while nonunique mutations were mostly associated with cancer status. These observations fit the hypothesis that the effects of aging are random and are not associated with outgrowth of particular cellular clones, while cancer patients are suffering from the effects of field cancerisation and that nonunique mutation frequency is capable of detecting at least some features of this process. Thus, HNSCC patients might harbour higher levels of clonal cell populations of D7S1482-mutant, but otherwise normal cells, than comparable controls. Further studies will be needed to prove such a relationship and if it may be used to assess cancer risk in general. Such studies will benefit from the use of additional hypermutable markers with reported high mutation levels, such as MycL1, DXS981 or UT5307.

Although our study illustrates the power of single-molecule analysis and confirms the essential role of aging in the accumulation of DNA damage, several limitations exist to firmly establish that an age-adjusted increase in microsatellite mutations is related to increased cancer risk. Most important is that, at least for the case of oral cavity carcinoma, the sampling site was also the site of a previous carcinoma. However, all patients were followed for a median time of 29 months after therapy to confirm the absence of recurrence and to assure that no cancer cells were present at the time of sampling. Another limitation is that a majority of HNSCC patients were treated with chemotherapy and/or radiation and such treatments may have confounded our findings. We therefore performed a subanalysis with patients whose only treatment was surgery and observed more significant effects than with the full set of cases. Another limitation of our study is that although we were able to match the large majority of cases and controls by age, sex and smoking histories, the demographics of HNSCC patients is greatly biased towards elderly males. The distribution of age differences was balanced between the cases and controls, but our study included more male smokers in the HNSCC case group than in the control group. In univariate analyses, females were less likely to demonstrate microsatellite mutations but since sex (and race) was not included in our initial hypothesis and to avoid overmatching our statistical model these two parameters were not included in the analysis. Future studies will need to be powered sufficiently to allow the inclusion of these, and other, variables.

A limitation of SP-PCR is that individual differences in the position of the germline allele sizes may lead to differences in the capability to detect mutations. For instance, in an individual with two closely spaced allele sizes that are only one repeat unit apart it is impossible to distinguish between the normally-sized larger allele and a mutant allele derived from the smaller allele by insertion of a single repeat unit. Our data predict that approximately 25% of mutations (20% single insertion and 5% single deletion) may be missed in such cases. More sophisticated statistical models may be capable of modeling the likelihood of these types of situations. Of the 56 subjects in our study, nine (16%) had D7S1482 allele sizes that were a single repeat unit apart but confounding is unlikely since they did not cluster in any specific group (five of 25 cases and six of 31 controls had closely spaced alleles). Our study was limited to the hypermutable marker D7S1482, which has one of the highest levels of mutations in upper aerodigestive tract cancers (Ahrendt *et al*, 2000; Xu *et al*, 2001) and the addition of newly identified hypermutable markers will increase the number of possible mutant alleles that can be scored.

For our study, we used DNAs that were isolated from cells collected through mouth rinse. Such sampling is noninvasive, can be done repeatedly and requires no special equipment. Unlike scraping or brushing of the oral cavity, mouth rinses are not restricted to specific sites of the oral cavity. Thus, in a subject with field cancerisation, mouth rinses may be more likely to sample multiple clonal cell populations that may be located at different subsites of the oral cavity. On the other hand, cells obtained from mouth rinse may already have gone through terminal differentiation with the associated degradation in DNA quality. It has also been reported that cells obtained by mouth rinse contain approximately 50% leukocytes, which may dilute the observed mutation levels. However, it was also reported that blood cells harbour many of the mutations we observed in cells obtained by mouth rinse (Osswald *et al*, 2003; Coolbaugh-Murphy *et al*, 2005). Future studies will be needed to establish differences between the various sampling techniques.

In conclusion, we describe a novel assay in clinical specimens based on direct amplification of single mutant alleles by SP-PCR. We demonstrated a strong correlation between microsatellite mutations and age and provide the first evidence that microsatellite mutations may be increased in non-cancerous cells obtained from oral rinses of HNSCC patients. Using this assay will open new avenues for the direct analysis of somatic mutational load in exposed cells obtained from easily accessible clinical specimens. These studies will be aided by the identification of additional hypermutable microsatellite markers and further automation of SP-PCR.

## Figures and Tables

**Table 1:** Characteristics of study subjects

	**Cases (*N*=25)**	**Controls (*N*=31)**	**Total (*N*=56)**
Age (median, range)	54 (31–73)	51 (36–82)	53 (31–82)
			
*Sex*			
Male	19	14	33
Female	6	17	23
			
*Race*			
White	22	30	52
Black	3	0	3
Other	0	1	1
			
*Tobacco use*			
Never	3	10	13
Former	6	11	17
Current	16	10	26
			
*Alcohol abuse* a			
Yes	4	2	6
No	21	29	50
			
*Tumour site*			
Oral cavity	10	N/A	N/A
Oropharynx	4		
Larynx	10		
Hypopharynx	1		
			
*Treatment modalities*			
Radiation therapy only	5	N/A	N/A
Chemotherapy only	1		
Radiation and chemotherapy	9		
Surgery only	10		

a Defined as more than three glasses per day.

**Table 2:** Multivariate analysis of overall, unique and nonunique mutations in D7S1482

	**Surgery only/controls (*N*=41)**	**Controls only (*N*=31)**	**All subjects (*N*=56)**
*Overall mutations*			
Age^a^	1.267, (0.991–1.618); 0.067	1.343 (1.117–1.614); **0.004**	1.340 (1.054–1.702); **0.021**
Pack-year^b^	0.968 (0.969–1.079); 0.561	0.959 (0.865–1.064); 0.441	0.990 (0.901–1.066); 0.636
Case status^c^	2.074 (1.174–3.665); **0.017**	N/A	1.609 (0.917–2.823); 0.104
			
*Unique mutations*			
Age^a^	1.163 (1.016–1.331); **0.035**	1.181 (1.019–1.369); **0.036**	1.240 (1.097–1.401); **0.002**
Pack-year^b^	0.987 (0.929–1.048); 0.666	0.978 (0.905–1.058); 0.585	0.989 (0.945–1.034); 0.624
Case status	1.183 (0.834–1.678); 0.352	N/A	1.116–0.835–1.493); 0.462
			
*Nonunique mutations*			
Age^a^	1.325 (0.962–1.824); 0.093	1.449 (1.154–1.819); **0.035**	1.394 (1.015–1.913); **0.045**
Pack-year^b^	0.960 (0.835–1.103); 0.567	0.948 (0.830–1.084); 0.443	0.975 (0.875–1.087); 0.654
Case status	2.645 (1.290–5.423); **0.012**	N/A	1.924 (0.915–4.048); 0.091

a Rate ratio for 10-year age interval (95% confidence interval); *P*-value GLM.

b Rate ratio for 10-pack-year interval (95% confidence interval); *P*-value GLM.

c Rate ratio for case status (95% confidence interval); *P*-value GLM.

Statistically significant values are indicated in bold.
